# PRESTO: Rapid calculation of order statistic distributions and multiple-testing adjusted P-values via permutation for one and two-stage genetic association studies

**DOI:** 10.1186/1471-2105-9-309

**Published:** 2008-07-13

**Authors:** Brian L Browning

**Affiliations:** 1 Department of Statistics, The University of Auckland, Auckland, New Zealand

## Abstract

**Background:**

Large-scale genetic association studies can test hundreds of thousands of genetic markers for association with a trait. Since the genetic markers may be correlated, a Bonferroni correction is typically too stringent a correction for multiple testing. Permutation testing is a standard statistical technique for determining statistical significance when performing multiple correlated tests for genetic association. However, permutation testing for large-scale genetic association studies is computationally demanding and calls for optimized algorithms and software. PRESTO is a new software package for genetic association studies that performs fast computation of multiple-testing adjusted P-values via permutation of the trait.

**Results:**

PRESTO is an order of magnitude faster than other existing permutation testing software, and can analyze a large genome-wide association study (500 K markers, 5 K individuals, 1 K permutations) in approximately one hour of computing time. PRESTO has several unique features that are useful in a wide range of studies: it reports empirical null distributions for the top-ranked statistics (i.e. order statistics), it performs user-specified combinations of allelic and genotypic tests, it performs stratified analysis when sampled individuals are from multiple populations and each individual's population of origin is specified, and it determines significance levels for one and two-stage genotyping designs. PRESTO is designed for case-control studies, but can also be applied to trio data (parents and affected offspring) if transmitted parental alleles are coded as case alleles and untransmitted parental alleles are coded as control alleles.

**Conclusion:**

PRESTO is a platform-independent software package that performs fast and flexible permutation testing for genetic association studies. The PRESTO executable file, Java source code, example data, and documentation are freely available at .

## Background

Permutation testing is often described as the gold-standard for determining statistical significance when performing multiple correlated tests for genetic association. Permutation testing can be applied to both case-control studies and trio studies (parents and affected offspring). In permutation testing, the case/control status of the individuals (for case-control studies) or the transmitted/untransmitted status of the parental chromosomes (for trio studies) are randomly permuted. The maximum test statistic, maximized over all tests for all markers, is calculated for the original affection/transmission status and for each permuted affection/transmission status. If *k *out of *P *permutations have a maximum test statistic greater than the maximum test statistic for the original data, the multiple-testing adjusted P-value for the experiment is (*k*+1)/(*P*+1) [1].

Permutation testing is computationally demanding for large-scale genetic association studies and requires an optimized software implementation. The PRESTO software package provides fast permutation testing for genome-wide association studies with thousands or millions of markers genotyped on thousands of samples. In addition to using permutation of the trait status to determine statistical significance of user-specified allelic and genotypic tests, PRESTO has three additional useful features: it can compute empirical distributions of order statistics so that the significance of sophisticated multi-marker statistics such as truncated products can be determined [2,3], it can perform stratified tests when sampled individuals are from multiple populations and each individual's population of origin is specified, and it can compute significance levels for two-stage genotyping designs using only first-stage genotyping data [4].

## Implementation

### Features

PRESTO is designed to be flexible and user-friendly. Input files have a simple format with rows corresponding to markers and columns corresponding to individuals (two columns per diploid genotype). This format is well-suited to large-scale genetic studies where there are typically many more markers (rows) than individuals (columns). Genetic marker data can be split up over multiple input files (e.g. one file per chromosome). There are no restrictions on how alleles or missing data are coded, and any sequence of non-white space characters can be used. Multi-allelic markers are permitted and are analyzed by creating a diallelic marker for each allele (grouping the other alleles) and testing each diallelic marker for association with the trait status. If the cases and controls are sampled from a stratified population and the strata are specified, PRESTO will automatically perform stratified allelic and genotypic tests [5,6] and will permute the trait status within each population stratum.

PRESTO can also compute significance levels of combined single locus and multi-locus analysis by representing clusters of haplotypes as diallelic markers as described in Browning and Browning [7]. By default, PRESTO permutes case-control status for individuals; however, it can also permute case-control status for chromosomes, so that transmitted/untransmitted data from trio studies (parents and affected offspring) can be analyzed.

PRESTO performs a Cochran-Mantel-Haenszel (CMH) test with continuity correction and a Mantel trend test [5,6]. The CMH test is a generalization to stratified data of the standard chi-square test of independence, and the Mantel trend test is a generalization to stratified data of the allelic trend test. When there is only one population stratum, these test statistics are equal to the standard test statistics after multiplying by *N*/(*N-*1) where *N *is the sample size. The CMH test is used to test for recessive, dominant, or overdominant effects. Although the CMH test can be used to test for allelic effects, the Mantel trend test is the preferred allelic test since it is robust to departure from Hardy-Weinberg equilibrium.

For each permutation of the trait status, PRESTO can store and report the top-ranked order statistics. The *j*-th order statistic is the *j*-th largest test statistic. For each marker, the test statistic is the largest allelic or genotypic test statistic for that marker. The empirical distributions of the top-ranked order statistics enable one to calculate an empirical P-value for any order statistic or any function of order statistics. An example of a function of order statistics is the *j*-th rank truncated product statistic, which is defined as the product of the nominal P-values of the *j *largest test statistics [2]. Rank truncated product statistics are useful for detecting association of multiple top-ranked markers with a trait, even when no individual marker has a significant multiple-testing adjusted P-value.

PRESTO can also calculate significance levels for two-stage genotyping designs from the first-stage genotype data using the technique described by Dudbridge [4]. In a two-stage genotype design, the sample is divided into two stages, a set of markers is genotyped in the first stage sample, and only the subset of first-stage markers with test statistics greater than a specified threshold are genotyped in the second stage sample. PRESTO samples the null distribution of the top-ranked order statistics for a two-stage genotyping design by using a subset of the first stage sample as a simulated first stage sample and the remainder of the first stage sample as a simulated second stage sample [4].

### Optimization techniques

PRESTO employs several techniques to optimize permutation testing on large-scale data sets. The permutations of the trait status are computed once and are stored. Each permutation of the trait status is represented as an array of Boolean (1 bit) variables in which the *k*-th binary indicator gives the affection status of the *k*-th chromosome in the input file. Each genetic marker is read once and is tested against all stored permutations of the trait status, so that only one marker is stored in memory at a time.

For each permutation of the trait status and each diallelic marker, a 2 × 3 contingency table is created where the rows are the cases and controls and the columns are the three possible genotypes. PRESTO obtains the 2 × 3 contingency table counts without having to check the permuted trait status and genotype for all individuals. Instead, PRESTO stores the indices of individuals with missing genotypes, heterozygote genotypes, and minor (least common) allele homozygote genotypes. The indices of individuals with major allele homozygote genotypes do not need to be stored because the case and control major allele homozygote genotype counts can be calculated from the case and control sample sizes, the case and control missing genotype counts, and the case and control heterozygote and minor allele homozygote genotype counts. For example, if there are *N *genotypes, and 90% of these are major allele homozygotes, then contingency tables for each permutation of the trait status are constructed by examining the permuted trait status of *N*/10 individuals instead of *N *individuals. This optimization is expected to be increasingly effective as denser marker sets are developed since markers with low minor allele frequency are much more numerous than markers with high minor allele frequency. An analogous optimization is used to obtain allele contingency table counts.

### Output files

PRESTO produces three output files: a log file, a P-value file, and a null distribution file. The log file summarizes the analysis and reports the command line parameters, the running time, and a list of all markers with a multiple-testing adjusted P-value less than 0.2.

The P-value file gives the chi-square test statistics for each allelic and genotypic test performed for each marker, and the permutation P-value for the maximum test statistic for each marker (maximized over all allelic and genotypic tests for the marker). If a marker has a maximum test statistic *t*_0 _when tested for association with the unpermuted trait status, and if for *k *out of *P *permutations of the trait status there exists at least one marker with a maximum test statistic ≥ *t*_0_, then the multiple-testing adjusted P-value for the marker is (*k*+1)/(*P*+1) [1]. The P-value file has a simple format and can be read into standard statistical software packages, such as R [8] for filtering and sorting. An R script for displaying QQ-plots of P-value distributions is available from the PRESTO web site.

The null distribution file gives the largest test statistics for each permutation of the trait status. If there are *P *permutations and the *K *largest test statistics are saved, then the null P-value file is a *P *× *K *white-space delimited matrix whose rows correspond to permutations and whose columns are the empirical distributions of the top-ranked order statistics. The entry in row *i *and column *j *is the *j*-th largest test statistic for the *i*-th permutation. Thus the *j*-th column gives the empirical distribution of the *j*-th largest test statistic.

## Results

### Computational time

Table 1 gives PRESTO running times for different scenarios when analyzing 449,446 autosomal markers genotyped in 2938 controls and 1749 Crohn's disease patients from the Wellcome Trust Case Control Consortium study [9]. The data were analyzed as a single population and as a stratified population with strata defined by the geographical origin of the samples within the United Kingdom. For the stratified analysis, the median stratum size was 584 individuals (range: 296 – 1542 individuals). For the two-stage genotyping scenarios, the empirical null distributions of the top-ranked order statistics were calculated assuming that only markers with a chi-square test statistic ≥ 10.0 (p = 0.0016) on an allelic, recessive, or dominant test were selected for genotyping in the second stage.

**Table 1:** PRESTO running times for the Wellcome Trust Case Control Consortium Crohn's disease study.

# order statistics	# strata	one-stage study	two-stage study
1	1	52.3 m	33.8 m
1	12	84.9 m	55.1 m
1000	1	56.6 m	34.3 m
1000	12	85.6 m	58.5 m

PRESTO computational times for 449,446 autosomal markers genotyped in 1749 cases and 2938 controls. Allelic trend test and dominant/recessive genotypic tests were performed using 1000 permutations of the trait status for 8 scenarios defined by the number of genotyping stages (1 or 2), the number of order statistic distributions calculated (1 or 1000), and the number of population strata (1 or 12). Running times were measured on an Intel Core 2 Duo processor E6600, 2.4 GHz processor with 4 GB of memory running Linux.

Running times for PRESTO 1.0.1 and PLINK 1.0 [10] were compared for this same data set on the same computer using a chi-square allelic test, 1000 permutations of the trait status, and a single population stratum. PRESTO was approximately 18 times faster than PLINK (50 minutes vs. 15 hours).

PRESTO's running time is linear in the number of samples, linear in the number of markers and linear in the number of permutations. Generally, 1000 permutations are sufficient to determine experiment-wide significance. PRESTO can also be run in parallel as described in the documentation.

### Memory requirements

Since only one marker is stored in memory at a time and since the trait status for each individual is stored using 2 bits, PRESTO's memory requirements are modest. If there are *P *permutations, *N *individuals, and the distributions of the top *K *order statistics are reported, then 2*NP*/8 bytes are allocated to store the permutations of the trait status and 8*KP *bytes are allocated to store the top-ranked order statistics (8 bytes per floating point number). In practice, 500 Mb of memory should suffice for values of *P *≤ 10,000, *K *≤ 2000, and *N *≤ 20,000.

## Discussion

Permutation testing with 1000 permutations of a large case-control genome-wide association study with 5000 individuals genotyped for 500,000 markers can be performed using PRESTO in approximately one hour of computing time (Table 1). With PRESTO, the costs of permutation testing (in terms of time and computing resources) are extremely low for many common study designs, and these costs compare very favourably to the costs associated with data generation (e.g. performing genotype assays, calling genotypes, and performing data quality control filtering).

There has been some debate regarding the number of permutations required. When performing *N *permutations, the smallest multiple-testing adjusted P-value one can observe is 1/(*N*+1) [1]. Thus, 1000 permutations can provide multiple-testing adjusted P-values as low as 0.001, which provide strong evidence of association. In the analysis of Wellcome Trust Case Control Consortium data described in Table 1, multiple-testing adjusted P-values of 0.05, 0.01, and 0.005 correspond to nominal P-values of 7.5 × 10^-8^, 1.5 × 10^-8^, and 6.2 × 10^-9 ^respectively. If additional permutations are desired, 10^4 ^or 10^5 ^permutations are easily performed on a large genome-wide data set like the WTCCC data set in Table 1, and even larger numbers of permutations can be easily performed for smaller studies (computation time is linear in the number of permutations).

Permutation testing is particularly appealing because of its simplicity. Recently, several more complex alternatives to permutation testing have been proposed [11-14]. These methods can be useful, more computationally efficient alternatives to permutation testing in some situations.

Some methods for computing adjusted P-values exploit the fact that for many common statistical tests, the correlated tests have an asymptotic multivariate normal distribution under the null hypothesis of no trait-marker correlation. Seaman and Müller-Myhsok have proposed estimating the asymptotic distribution and sampling directly from it [14], and Conneely and Boehnke have proposed estimating the asymptotic distribution and calculating probabilities under this distribution using numerical integration [11]. Either approach can be used to estimate the probability of observing a minimum P-value smaller than the observed minimum P-value. Both approaches are particularly well-suited to situations where covariate data are available or multiple quantitative phenotypes are tested. When the asymptotic distribution is accurately estimated, these methods are shown to give accurate results (compared to permutation as the gold standard) for candidate gene studies.

There are some limitations with these approaches that estimate the asymptotic multivariate normal distribution of the test statistics. These methods do not estimate significance levels for two-stage genotyping designs. A more severe restriction is that these methods are typically limited to several hundred correlated tests. Seaman and Müller-Myhsok and Conneely and Boehnke suggest that the number of samples should be at least 10 times the number of tests performed in order to accurately estimate the asymptotic multivariate normal distribution [11,14]. So these methods cannot be directly applied to hundreds of thousands of single marker tests in a genome-wide association study.

Other alternatives to permutation testing are based on importance sampling. Kimmel and Shamir [13] have proposed a method that uses importance sampling to accurately estimate extremely small multiple-testing adjusted P-values, and Kimmel and colleagues [12] have modified this method to work with data from a stratified population. Decay of linkage disequilibrium with increasing genomic distance is exploited to further improve the computational efficiency of these methods.

These importance sampling methods lack some of the features that are found in PRESTO. The methods do not calculate significance for two-stage genotyping designs, and they do not calculate adjusted P-values for general order statistics. In the extension to stratified data, the association test statistic used in Kimmel et al [12] will have suboptimal power because it ignores the population structure of the data (the population structure is incorporated in the importance sampling, but not in the test statistic). The method of Kimmel and colleagues [12] can be modified to use a test statistic for stratified data (such as those used in PRESTO), but this would dramatically increase the computation time because their method loops through all possible contingency tables for each sampled permutation, and the number of contingency tables consistent with a permutation increases exponentially with the number of population strata.

Methods for computing multiple-testing adjusted P-values that are based on asymptotic multivariate normal distributions or importance sampling, are more complex than permutation testing, and require the asymptotic approximations to be accurate. In addition, when testing a single binary trait, these alternative methods provide little or no decrease in computational time relative to permutation testing with PRESTO, unless one is performing more than 1000 permutations.

## Conclusion

PRESTO is a flexible, platform-independent software package that determines multiple-testing adjusted statistical significance for large-scale genetic association studies by using permutation of the trait status. PRESTO is faster than existing permutation testing software and can analyze a large genome-wide association study (500 K markers, 5 K individuals, 1 K permutations) in approximately one hour of computing time. PRESTO can be used with stratified data from multiple populations and with two-stage genotyping designs. PRESTO can also report empirical null distributions for the top-ranked statistics (i.e. order statistics) so that statistical significance can be determined for any test statistic calculated in terms of order statistics.

## Availability and requirements

• **Project name: **PRESTO

• **Project home page: **

• **Operating system(s): **Platform independent

• **Programming language: **Java

• **Other requirements: **standard edition (SE) Java Runtime Environment (JRE) 5.0 (or higher)

• **License: **freely available for academic and commercial use.
